# Ferulic Acid Derivatives Ameliorate Intestine Barrier Destruction by Alleviating Inflammatory Responses in Dextran Sulfate Sodium-Induced Inflammatory Bowel Disease

**DOI:** 10.3390/toxics12040268

**Published:** 2024-04-03

**Authors:** Yeon-Yong Kim, Gayeong Hur, Hyun-Jae Jang, Seungwon Jeong, Seung Woong Lee, Seung-Jae Lee, Mun-Chual Rho, Sang-Hyun Kim, Soyoung Lee

**Affiliations:** 1 Functional Biomaterials Research Center, Korea Research Institute of Bioscience and Biotechnology (KRIBB), Jeongeup 56212, Republic of Korea; gyy123@kribb.re.kr (Y.-Y.K.); gayeong1115@gmail.com (G.H.); jsw0212@kribb.re.kr (S.J.); lswdoc@kribb.re.kr (S.W.L.); seung99@kribb.re.kr (S.-J.L.); rho-m@kribb.re.kr (M.-C.R.); 2 Natural Product Research Center, Korea Research Institute of Bioscience and Biotechnology (KRIBB), Cheongju 28116, Republic of Korea; water815@kribb.re.kr; 3 Cell and Matrix Research Institute, Department of Pharmacology, School of Medicine, Kyungpook National University, Daegu 41944, Republic of Korea

**Keywords:** inflammatory bowel disease, dextran sulfate sodium, ferulic acid, *Portulaca oleracea* L.

## Abstract

Inflammatory bowel disease (IBD), a chronic disorder affecting the colon and rectum, involves the overproduction of pro-inflammatory cytokines causing damage to tight junctions (TJ) in the intestinal epithelial cells and chronic inflammation. The current mainstay of treatment, sulfasalazine, often causes adverse effects, thereby necessitating the exploration of alternative herbal medicines with fewer side effects. *Portulaca oleracea* L. (*P. oleracea*), a traditional medicinal herb, contains feruloyl amide compounds. We synthesized new compounds by conjugating ferulic acid (FA) with (±)-octopamine. Our study focused on novel FA derivatives that demonstrate protective effects against the intestinal epithelial barrier and inflammatory responses. In lipopolysaccharide-induced cells, C1 and C1a inhibited the production of inflammatory mediators. In Caco-2 cells, these compounds maintained the TJ protein expression, thereby demonstrating their protective effects on the epithelial barrier. In a mouse model of dextran sulfate sodium-induced IBD, a treatment with these compounds ameliorated features including a body weight reduction, colon shortening, an increased disease activity index, and histopathological changes. Furthermore, C1a demonstrated greater efficacy than C1 at the same concentration. These findings suggest that the novel FA derivative (C1a) effectively alleviates clinical signs and inflammatory mediators in IBD, making these compounds potential candidates as natural medicines for the treatment of IBD.

## 1. Introduction

Inflammatory bowel disease (IBD) is a chronic disease of unknown etiology. Although the severity of IBD in Asia remains lower than in the West, its global incidence and prevalence rates are increasing [1,2]. IBD comprises ulcerative colitis and Crohn’s disease, which are differentiated by the location of the inflammatory lesions. Ulcerative colitis affects the rectum and colon, whereas CD manifests from the mouth to the anus; both conditions are accompanied by inflammatory responses at the lesion site [3]. During disease progression in the colon, the mucosal epithelial barrier is compromised and macrophages secrete chemokines and pro-inflammatory cytokines [4,5]. Patients with IBD exhibit elevated pro-inflammatory cytokine production and immune dysregulation. In these patients, intestinal immune cells and macrophages have been found to secrete large amounts of pro-inflammatory cytokines, such as tumor necrosis factor (TNF)-α, interleukin (IL)-1β, and IL-6, leading to damage of the intestinal epithelial monolayers and subsequently the mucosal layer [6]. Therefore, targeting these inflammatory responses has become a focal point in therapeutic development.

Macrophages are integral to immune and adaptive responses and play a crucial role in the maintenance of inflammation and homeostasis [7]. Activated by external stimuli, such as lipopolysaccharide (LPS), macrophages initiate transcription factors that regulate inflammatory reactions, leading to the expression of enzymes, such as inducible nitric oxide synthase (iNOS) and cyclooxygenase-2 (COX-2). This cascade results in the production of inflammatory mediators, such as nitric oxide (NO), prostaglandin E2, and inflammatory cytokines [8]. While these inflammatory mediators play a major role in killing bacteria or eliminating antigens in the early stages of immune responses, the excessive formation of inflammatory mediators can induce inflammatory diseases and tissue remodeling [9]. Given that IBD involves hyperactivated macrophages, they are considered a potential therapeutic target [10].

Persistent inflammatory responses induce a loss of cell-to-cell tight junction (TJ) molecules, such as zonula occludens (ZO)-1 and occludin, resulting in disruption of the intestinal barrier structure. The intestinal structure consists of the crypt and lamina propria, which are protected by a layer of epithelial cells strongly adhered to by TJ molecules [11]. In the healthy intestine, TJ molecules maintain the movement of nutrients and the permeability of molecules through the paracellular space and play a defensive role against inflammation. In contrast, TJ degradation during disease increases the permeability and uptake of intestinal microbial antigens and pathogens [12]. In clinical studies, patients with IBD have shown reduced expression levels of TJ-associated proteins and increased intestinal permeability due to a compromised epithelial barrier function. Consequently, pathogens and endotoxins enter the circulation, resulting in endotoxemia and organ dysfunction [13,14]. In addition, patients have been reported to exhibit enhanced pro-inflammatory cytokine production and immune dysregulation [15]. Thus, the regulation of inflammatory responses in IBD can be expected to maintain TJ levels and preserve the intestinal structure, thereby ameliorating intestinal diseases. 

The existing drugs for symptomatic treatment of IBD include immunosuppressants, anti-biotics, biologics, and anti-inflammatory drugs [16,17]. Sulfasalazine (SS), an aminosalicylic-acid-based drug, is widely used for mild-to-moderate IBD but has limited efficacy and associated adverse effects, including nausea, vomiting, dyspepsia, diarrhea, headache, pancreatitis, agranulocytosis, and pulmonary toxicities [18,19]. Recent studies have focused on developing IBD treatments using plant extracts with fewer side effects [20,21]. *Portulaca oleracea* L. (*P. oleracea*), an annual plant, has been reported to have anti-cancer, anti-oxidant, nicotine removal, muscle relaxant, and anti-microbial properties. Additionally, this plant has been used to treat gastroenteritis, diarrhea, dysentery, skin ulcers, inflammation, and other diseases [22,23,24]. Our previous studies have shown that ethanol extracts of *P. oleracea* regulate the production of inflammatory factors in mouse macrophages and inhibit IBD symptoms [25]. Ferulic acid (4-hydroxy-3-methoxy cinnamic acid) (FA), a major polyphenol compound in *P. oleracea*, possesses anti-oxidant and anti-inflammatory properties [26] and has the potential to treat various diseases, such as cardiovascular diseases, Alzheimer’s disease, and diabetes [27,28,29]. FA has demonstrated protective effects on the intestinal epithelial barrier by significantly increasing the expression of TJ-related proteins such as claudin-1, occludin, and zonula occludens (ZO)-1 in human intestinal epithelial cells [11,30]. Although FA may have protective effects against various inflammatory symptoms in IBD, its clinical application is limited because of its poor pharmacokinetic properties and low bioavailability [31,32]. Therefore, we synthesized the new compounds by reacting FA and (±)-octopamine, and expect the improvement to have a beneficial effect. In the present study, we evaluated the in vivo and in vitro anti-inflammatory effects of FA derivatives on IBD.

## 2. Materials and Methods

### 2.1. Synthesis and Isolation of the Ferulic Acid Derivative 4-O-(E)-Feruloyl-N-feruloyloctopamine

#### 2.1.1. General Experimental Procedures

The synthetic mixtures were purified using flash column chromatography (CombiFlash RF, Teledyne Isco, Lincoln, NE, USA). The thin-layer chromatography (TLC) was performed using silica gel 60 F_254_ and RP-18 F_254s_ (Merck, Darmstadt, Germany)-precoated plates under UV light (254 nm). The infrared (IR) spectra were obtained using a Jasco-4600 FT-IR instrument (Jasco Corp., Tokyo, Japan). The ^1^H and ^13^C NMR spectra were recorded using a JEOL JNM-ECA600 instrument (JEOL, Tokyo, Japan). The ESI-MS data were obtained using an Agilent 1100 series MSD mass spectrometer (Agilent Technologies, Wilmington, DE, USA) and Waters SYNAPT G2-Si HDMS mass spectrophotometer (Waters, Milford, MA, USA). All reagents were purchased from Sigma-Aldrich (St. Louis, MO, USA). 

#### 2.1.2. Preparation of *N-(E)*-Feruloyloctopamine Derivatives (C1 and C1a)

A mixture of (±)-octopamine HCl (1.0 g, 5.2 mmol), ferulic acid (0.88 g, 4.5 mmol), and *N*,*N*-dimethylformamide (12 mL) was stirred at room temperature. To the dissolved solution, etylene dichloride (1.6 mL, 9.0 mmol) and triethylamine (0.8 mL) were stirred at −5 °C for 10 min. The mixture was stirred overnight at room temperature. After completion of the reaction, the solvent was evaporated and the residue was separated via flash column chromatography using a silica column (SiO_2_, 120 g; CHCl_3_:MeOH 1:0 → 30:1, *v*/*v*). Fractions 9 (363.6 mg) and 7 (81.6 mg) were identified as *N*-*trans*-feruloyloctopamine derivatives (C1 and C1a) using ^1^H, ^13^C, and 2D NMR (COSY, HMQC, HMBC, and NOESY, respectively) and MS spectroscopic data.

#### 2.1.3. *N-(E)*-Feruloyloctopamine (C1)

Yield 19%; White solid; ESI-MS: *m/z* 328.1 [M–H]^−^; ^1^H NMR (600 MHz, methanol-*d_4_*) *δ*_H_: 7.44 (1H, d, *J* = 15.6 Hz, H-7), 7.22 (2H, d, *J* = 8.4 Hz, H-2′, 6′), 7.12 (1H, d, *J* = 1.8, H-2), 7.02 (1H, dd, *J* = 7.8, 1.8 Hz, H-6), 6.79 (1H, d, *J* = 7.8, H-5), 6.77 (2H, d, *J* = 8.4 Hz, H-3′, 5′), 6.46 (1H, d, *J* = 15.6 Hz, H-8), 4.72 (1H, dd, *J* = 7.8, 4.8 Hz, H-7′), 3.53 (1H, dd, *J* = 13.2, 4.8 Hz, H-8′a), 3.44 (1H, dd, *J* = 13.2, 7.8 Hz, H-8′b), 3.88 (3H, s, OCH_3_-3); ^13^C NMR (150 MHz, methanol-*d_4_*) *δ*_C_: 169.6 (C-9), 158.3 (C-4′), 150.0 (C-4), 149.4 (C-3), 142.4 (C-7), 134.9 (C-1′), 128.6 (C-2′, 6′), 128.4 (C-1), 123.4 (C-6), 118.8 (C-8), 116.6 (C-5), 116.3 (C-3′, 5′), 111.7 (C-2), 73.6 (C-7′), 48.5 (C-8′).

#### 2.1.4. 4-*O-(E)*-Feruloyl-*N-(E)*-feruloyloctopamine (C1a)

Yield 4%; White solid; HR-ESI-MS: *m*/*z* 504.1665 [M − H]^−^; ^1^H NMR (600 MHz, DMSO-*d_6_*) *δ*_H_: 9.70 (1H, s, OH-4″), 9.26 (1H, s, OH-4′), 8.06 (1H, t, *J* = 6.0 Hz, NH), 7.74 (1H, d, *J* = 15.6 Hz, H-7″), 7.43 (1H, s, H-2″), 7.41 (1H, d, *J* = 15.6 Hz, H-7), 7.33 (1H, s, H-2), 7.21 (1H, dd, *J* = 7.8, 1.8 Hz, H-6″), 7.16 (2H, m, H-5, 6, overlap), 7.15 (1H, d, *J* = 8.4 Hz, H-2′, 6′), 6.82 (1H, d, *J* = 8.4, H-5″), 6.74 (1H, d, *J* = 16.2 Hz, H-8″), 6.72 (2H, d, *J* = 8.4 Hz, H-3′, 5′), 6.72 (1H, d, *J* = 16.2 Hz, H-8), 5.35 (1H, d, *J* = 4.2 Hz, OH-7′), 4.56 (1H, m, H-7′), 3.40 (1H, m, H-8′a), 3.21 (1H, m, H-8′b), 3.84 (3H, s, 3″-OCH_3_), 3.81 (3H, s, 3-OCH_3_); ^13^C NMR (150 MHz, DMSO-*d_6_*) *δ*_C_: 165.0 (C-9), 164.6 (C-9″), 156.4 (C-4′), 151.2 (C-3), 149.8 (C-4″), 147.9 (C-3″), 147.1 (C-7″), 140.2 (C-4), 137.9 (C-7), 133.9 (C-1′), 133.8, (C-1), 127.1 (C-2′, 6′), 125.4 (C-1″), 123.7 (C-6″), 123.4 (C-5), 122.6 (C-8), 120.0 (C-6), 115.5 (C-5″), 114.7 (C-3′, 5′), 113.0 (C-8″), 111.4 (C-2′, C-2″, overlap), 71.1 (C-7′), 47.0 (C-8′), 55.7 (OCH_3_-3, 3″, overlap).

### 2.2. Cell Culture

J774A.1 (ATCC^®^ TIB-67™) cells, a mouse macrophage cell line, were cultured in DMEM (Gibco, Grand Island, NY, USA) supplemented with 10% non-heat-inactivated fetal bovine serum (FBS, Sigma-Aldrich) and 1% penicillin and streptomycin. Caco-2 (ATCC^®^ HTB-37™) cells, a human intestinal epithelial cell line, were cultured in DMEM supplemented with 10% heat-inactivated FBS and 1% penicillin and streptomycin. The cells were cultured in a humidified 5% CO_2_ atmosphere at 37 °C.

### 2.3. Isolation of Mouse Peritoneal Macrophage

The mouse peritoneal macrophages were isolated 4 days after an intraperitoneal injection of 3 mL thioglycollate in the mice. After sacrificing the mice, 5 mL of phosphate-buffered saline (PBS) was injected into the peritoneum followed by a gentle massage of the abdomen. To isolate the peritoneal cells, the peritoneal cavity was carefully opened and the fluid was collected using a Pasteur pipette. The fluid was centrifuged at 1900× *g* for 10 min at 25 °C and the supernatant was discarded. The isolated mouse peritoneal macrophages were resuspended in 1 mL of DMEM. The isolated cells were immediately counted using a hemocytometer and seeded for further analyses.

### 2.4. Cell Viability

The J774A.1 cells were seeded in 96-well plates at a density of 5 × 10^4^ cells/100 μL of media on each well and allowed to stand overnight, and then the cells were treated with 10 and 20 μM of FA, C1, and C1a. The Caco-2 cells were seeded in 24-well plates at a density of 1.5 × 10^5^ cells/500 μL of media on each well and allowed to stand overnight, and then the cells were treated with 20 μM of C1 and C1a. After 24 h, the cells were exposed to 1 mg/mL of MTT solution (3-(4,5-dimethylthiazol-2-yl)-2,5-diphenyltetrazolium bromide) for 2 h at 37 °C. Following the removal of the supernatant, the formazan byproduct in the cells was dissolved in dimethyl sulfoxide (DMSO, Sigma-Aldrich) and the absorbance was measured at 570 nm using a microplate reader (Thermo-Fisher Scientific, Waltham, MA, USA).

The mouse peritoneal macrophages were seeded in 96-well plates at a density of 1 × 10^5^ cells/100 μL of media on each well and allowed to stand overnight, and then the cells were treated with 20 μM of FA, C1, and C1a. After 24 h, the cells were treated with 20 μL of EZ-Cytox solution (WST-1, Dogen, Suwon, Korea) and incubated for 4 h at 37 °C. The absorbance was measured at 450 nm using a microplate reader (Thermo Fisher Scientific).

### 2.5. NO Assay

The J774A.1 cells were seeded in 96-well plates at a density of 5 × 10^4^ cells/100 μL of media on each well and allowed to stand overnight. The cells were treated with 10 and 20 μM of FA, C1, and C1a for 1 h and then stimulated with 100 ng/mL of LPS for 24 h. 

The mouse peritoneal macrophages were seeded in 96-well plates at a density of 1 × 10^5^ cells/100 μL of media on each well and allowed to stand overnight. The cells were treated with 20 μM of FA, C1, and C1a and then treated with 1 μg/mL of LPS for 24 h. The levels of NO in the culture media were determined using a nitric oxide detection kit (iNtRON Biotechnology, Seongnam, Korea). The absorbance was recorded at 540 nm using a microplate reader (Thermo Fisher Scientific).

### 2.6. Quantitative Polymerase Chain Reaction (qPCR)

The J774A.1 cells were seeded in 12-well plates at a density of 5 × 10^5^ cells/1 mL of media in each well and allowed to stand overnight. The cells were treated with 10 μM of quercetin and 10 and 20 μM of C1 and C1a for 1 h and then stimulated with 100 ng/mL of LPS for 6 h. The Caco-2 cells were seeded in 24-well plates at a density of 5 × 10^5^ cells/500 μL of media on each well and allowed for overnight. The cells were treated with 10 μM of C1 and C1a for 1 h and then stimulated with 25 ng/mL of TNF-α/IL-1β for 24 h. To determine the RNA expression in the descending colon of mice with IBD, 5 g colon tissue samples were minced into small pieces. Cellular and tissue RNA samples were isolated using an Ambion RNA isolation kit (Thermo Fisher Scientific) according to the manufacturer’s protocol. The first-strand complementary DNA (cDNA) was prepared from 1 μg of RNA using a PrimeScript™ 1st Strand cDNA synthesis kit (Takara, Shiga, Japan). The qPCR was performed using a Thermal Cycler Dice Real-Time System (Analytik Jena, Jena, Germany) with TB Green™ Premix Ex Taq (TaKaRa) according to the manufacturer’s protocol. The relative quantification of the mRNA expression was achieved using qPCR software (qPCRsoft 3.0, Analytik Jena). The primer sequences are listed in Table S1.

### 2.7. Western Blotting

The J774A.1 cells were seeded in 6-well plates at a density of 1 × 10^6^ cells/1 mL of media in each well and allowed to stand overnight. The cells were treated with 10 μM of quercetin and 20 μM of C1 and C1a for 1 h and then stimulated with 100 ng/mL of LPS for 24 h. The Caco-2 cells were seeded in 6-well plates at a density of 2 × 10^6^ cells/1 mL of media on each well and allowed to stand overnight. The cells were treated with 10 μM of C1 and C1a for 1 h and then stimulated with 25 ng/mL of TNF-α/IL-1β for 24 h. The total protein was isolated using a cell lysis buffer (Cell Signaling Technology, Danvers, MA, USA) supplemented with a protease/phosphatase inhibitor cocktail (Thermo Fisher Scientific) according to the manufacturer’s protocol. The protein amounts were quantified using a Bradford protein assay kit (Bio-Rad Laboratories, Hercules, CA, USA). Equal amounts of protein were separated by 4–12% sodium dodecyl sulfate–polyacrylamide gel electrophoresis and transferred onto polyvinylidene membranes. After the non-specific sites of the proteins on the membrane were blocked with 5% bovine serum albumin (BSA) for 1 h, the membrane was incubated with a specific primary antibody overnight at 4 °C and then incubated with a secondary antibody for 1 h at 25 °C. The antibodies against iNOS (#13120S, rabbit monoclonal, 1:1000), COX-2 (#4842S, rabbit polyclonal, 1:1000), ZO-1 (#13663, rabbit monoclonal, 1:1000), occludin (#3704S, rabbit monoclonal, 1:1000), and β-actin (#4967S, rabbit monoclonal, 1:1000) were obtained from Cell Signaling Technology. The immunoblot signals were visualized using a chemiluminescent substrate kit (iNtRON Biotechnology) and a ChemiDoc MP imaging system (Bio-Rad Laboratories).

### 2.8. Enzyme-Linked Immunosorbent Assay (ELISA)

The phosphorylation of nuclear factor (NF)-κB in LPS-stimulated J774A.1 cells was determined with an ELISA kit (#7834S; Cell Signaling Technology) according to the manufacturer’s protocol. The J774A.1 cells were seeded in 6-well plates at a density of 1 × 10^6^ cells/1 mL of media in each well and allowed to stand overnight. The cells were treated with 10 μM of quercetin and 20 μM of C1 and C1a for 1 h and then stimulated with 100 ng/mL of LPS for 1 h. The proteins were isolated using a cell lysis buffer (Cell Signaling Technology).

The levels of serum myeloperoxidase (MPO; R&D Systems, Minneapolis, MN, USA), C-reactive protein (CRP; R&D Systems), immunoglobulin (Ig) A (RayBiotech, Norcross, GA, USA), and IgG2a (BD Biosciences, San Diego, CA, USA) were measured using ELISA according to the manufacturer’s protocol. Following sacrifice, blood was collected from the mice via cardiac puncture. To isolate the serum, the blood samples were allowed to clot at 25 °C for 1 h and then centrifuged at 2200× *g* and for 15 min at 4 °C. The supernatant was collected and stored at −80 °C immediately until analysis. 

### 2.9. Animals

The seven-week-old male ICR mice were obtained from Orient Bio (Gwangju, South Korea). The forty-nine mice were randomly divided into seven groups. The mice were housed in the animal room under 12 h dark/light cycles and at a constant temperature of 20 ± 5 °C, and were fed *ad libitum* with standard laboratory chow. The animal experiments were approved by the Ethical Committee for Animal Care and Use and the Institutional Animal Care and Use Committee of the Korea Research Institute of Bioscience and Biotechnology.

### 2.10. Induction of IBD Mouse Model

Dextran sulfate sodium (DSS)-induced IBD mouse models are commonly used to evaluate the efficacy of drugs against intestinal inflammation. In addition, DSS is considered an intestinally toxic substance because it induces epithelial damage and TJ destruction [33]. The animal experiment consisted of seven groups followed by a normal control, DSS administration, DSS administration with 50 mg/kg of SS, DSS administration with 1 or 5 mg/kg of C1, and DSS administration with 1 or 5 mg/kg of C1a. The mice in the DSS group received drinking water containing 3% DSS for 14 days. The mice assigned to the SS, C1, and C1a groups received daily oral administration throughout the experimental period. All compounds were dissolved in PBS. The mice in the control group were provided drinking water throughout the experimental period. Throughout the experiment, the body weight and disease activity index (DAI) were measured daily. The DAI indicates the degree of inflammatory response in mice and was estimated according to the standards shown in Table S2. At the end of the experiment, all mice were sacrificed and the large intestines were separated from the vermiform appendix up to the anus.

### 2.11. Intestine Tissue Processing for Histopathology

For the histological analysis, approximately 0.3–0.5-cm-thick sections of the descending colon were excised and fixed immediately in 4% neutral formalin. After fixation, the tissues were washed with running water to remove the formalin. The tissue sections underwent further processing, including dehydration, clearing, and paraffin wax infiltration using a TP1020 automatic benchtop tissue processor (Leica Biosystems, Wetzlar, Germany). Following processing, the tissue samples were embedded in paraffin wax blocks, sectioned at 5 μm thickness, and adhered onto poly-L-lysine-coated slides.

### 2.12. Immunohistochemical (IHC) Staining

The IHC staining was performed using an Envision Detection Kit (#5007; DAKO, Glostrup, Denmark). The paraffin-embedded tissue sections were deparaffinized using xylene and rehydrated in graded concentrations of ethanol (100%, 90%, 80%, and 70%). Following deparaffinization and washing with distilled water, the tissue sections were incubated with primary antibodies against TNF-α (ab6671, Abcam, Cambridge, UK) IL-1β (ab9722, Abcam), and IL-6 (ab6672) for 12 h at 4 °C. The tissue sections were incubated with horseradish-peroxidase-conjugated secondary antibodies for 30 min. The immunoreactivity was visualized using the chromogen diaminobenzidine and the sections were examined under a photomicroscope (Olympus, Tokyo, Japan) at ×200 magnification.

### 2.13. Hematoxylin and Eosin (H&E) Staining

Following deparaffinization and washing with distilled water, the tissue sections underwent hematoxylin (Sigma-Aldrich) staining for nuclear counterstaining and subsequent eosin (Sigma-Aldrich) staining for cytoplasmic visualization. The slides were rinsed thoroughly with distilled water after each staining step. The slides were dehydrated in a graded concentration of ethanol from 70 to 100% and were mounted using Synthetic Mountant™ (Thermo Fisher Scientific). Images of the tissue sections were captured under a microscope (Olympus) at ×100 magnification.

### 2.14. Statistical Analysis

All data are presented in the graphical format as the mean ± SEM of independent experiments. For the SEM, we used the calculation method of dividing the standard error by the square root of the sample size. All statistical analyses were performed using Prism, version 7 (GraphPad Software, San Diego, CA, USA). A one-way analysis of variance (ANOVA) followed by Dunnett’s test was performed. Here, a *p*-value of <0.05 was considered statistically significant.

## 3. Results

### 3.1. C1 and C1a Inhibited LPS-Induced Inflammatory Responses in Mouse Macrophages

The FA derivatives *N*-(*E*)-feruloyloctopamine (C1) and 4-*O*-(*E*)-feruloyl-*N*-(*E*)-feruloyloctopamine (C1a) were synthesized in our study of a novel therapeutic agent for IBD, as shown in Figure S1. Their chemical structures were determined using spectroscopic data, such as ^1^H, ^13^C, COSY, HMQC, HMBC, NOSEY NMR, and HRESIMS data (Figures S2–S13). The chemical structures of compounds C1 and C1a showed the presence of an amide linkage between the octopamine and the *trans*-feruloyl moiety based on the HMBC correlation of H-8′ (*δ*_H_ 3.40, 1H, m, H-8′a; 3.21, 1H, m, H-8′b) and C-9 (*δ*_C_ 165.0). The C1 isolated from our previously study was identified as *N*-(*E*)-feruloyloctopamine by comparison with NMR and MS spectral data from the literature [34].

The HR-ESI-MS data for C1a were detected as a deprotonated ion peak at *m*/*z* 504.1665 ([M − H]^−^, calculated as 504.1658) and its molecular formula was elucidated as C_28_H_27_NO_8_. The NMR spectroscopy data for C1a indicated the presence of an additional *trans*-feruloyl moiety (*δ*_H_: 9.70 (1H, s, OH-4″), 7.74 (1H, d, *J* = 15.6 Hz, H-7″), 7.43 (1H, s, H-2″), 7.21 (1H, dd, *J* = 7.8, 1.8 Hz, H-6″), 6.82 (1H, d, *J* = 8.4, H-5″), 6.74 (1H, d, *J* = 16.2 Hz, H-8″), 3.84 (3H, s, 3″-OCH_3_); *δ*_C_: 164.6 (C-9″), 149.8 (C-4″), 147.9 (C-3″), 147.1 (C-7″), 125.4 (C-1″), 123.7 (C-6″), 115.5 (C-5″), 113.0 (C-8″), 111.4 (C-2″, overlap), 55.7 (OCH_3_- 3″, overlap)), and the nuclear Overhauser effect (NOE) correlation between OCH_3_-3 and H-7″ suggests that the second feruloyl group is located close to the OH-4 of the original feruloyl moiety. Its NMR data were similar to those of hibiscuwanin A [35], except for the presence of the additional hydroxy group at C-7′ (*δ*_C_ 71.1) in the C1a compound. The H-7′ [*δ*_H_ 4.56 (1H, m, H-7′)] signal of C1a was deshielded compared to that of hibiscuwanin A, in which the feruloyl and tyramine moieties are linked by an amide bond, and was correlated with the aromatic resonances at C-2′, -6′ (*δ*_C_ 127.1) in the HMBC spectrum. Therefore, the novel compound C1a was elucidated as 4-*O*-(*E*)-feruloyl-*N*-(*E*)-feruloyloctopamine.

**Figure 1:**
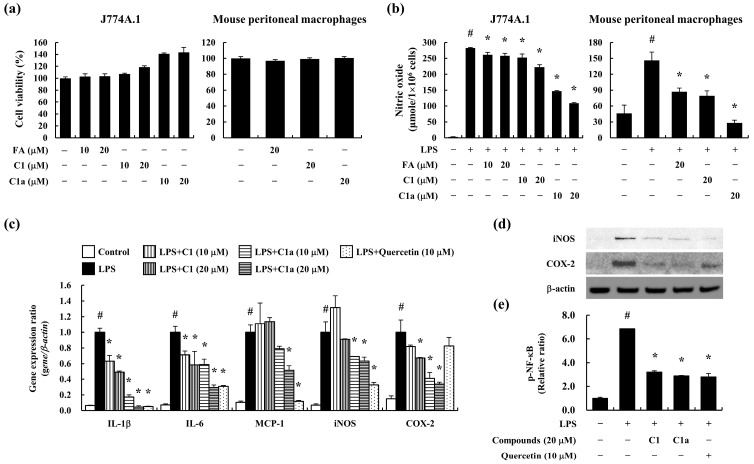
Anti-inflammatory effect of FA derivatives C1 and C1a in macrophages: (**a**) the cytotoxicity of FA, C1, and C1a was determined on J774A.1 cells and mouse peritoneal macrophages; (**b**) LPS-stimulated nitric oxide production was determined using a nitric oxide detection kit on J774A.1 cells and mouse peritoneal macrophages; (**c**) the LPS-stimulated expression of pro-inflammatory cytokines and mediators including IL-1β, IL-6, MCP-1, iNOS, and COX-2 in J774A.1 cells was analyzed by qPCR; (**d**) the LPS-stimulated activation of intracellular mediators, including iNOS and COX-2, was confirmed by Western blot. (**e**) the LPS-stimulated phosphorylation of NF-κB was assessed by ELISA. Data are means ± SEM of three independent experiments. Note: ^#^ *p* < 0.05 is a significant difference compared with control group; * *p* < 0.05 is a significant difference compared with LPS-treated group.

To assess the cytotoxicity of C1 and C1a in vitro, we used the J774A.1 cells and mouse peritoneal macrophages. The pre-treatment of FA, C1, and C1a did not show cytotoxicity up to 20 μM in concentration on J774A.1 cells and mouse peritoneal macrophages (Figure 1a). FA was used as the positive control.

To determine the anti-inflammatory effects of C1 and C1a, the J774A.1 cells and mouse peritoneal macrophages were stimulated with LPS after pre-treatment with C1 and C1a for 1 h. The LPS-stimulated J774A.1 cells and mouse peritoneal macrophages secreted high levels of NO, which were suppressed by a pre-treatment with C1 and C1a. In particular, C1a showed greater inhibitory efficacy than FA or C1 (Figure 1b).

The activated macrophages highly expressed the pro-inflammatory cytokines and mediators such as IL-1β, IL-6, monocyte chemoattractant protein (MCP)-1, iNOS, and COX-2. The LPS-stimulated J774A.1 cells expressed high levels of pro-inflammatory cytokines and mediators, which were suppressed by the pre-treatment with C1 and C1a (Figure 1c). Furthermore, iNOS and COX-2 are inflammatory mediators induced by NF-κB, resulting in inflammation [7]. The LPS-stimulated J774A.1 cells showed an increase in iNOS and COX-2 levels and phosphorylation of NF-κB but were decreased by the pre-treatment of C1 and C1a (Figure 1d,e). These results suggest that C1 and C1a inhibited the pro-inflammatory mediators by suppressing the activation of NF-κB.

### 3.2. C1 and C1a Inhibited the Symptoms of IBD in DSS-Induced IBD Mice Model

**Figure 2:**
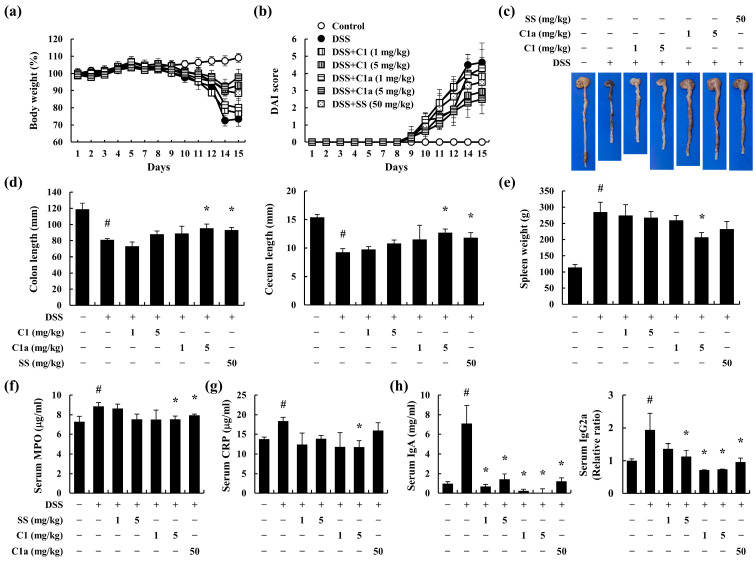
Efficacy of the FA derivatives C1 and C1a in a DSS-induced IBD mice model. To investigate the efficacy of C1 and C1a, we established an IBD mice model through the administration of 3% DSS for 14 days. SS, C1, and C1a were administered to mice daily. The DSS-induced loss of body weight (**a**); elevation of the DAI (**b**); shortness of the intestine (**c**), including the colon and cecum (**d**); and increase in spleen weight (**e**) were ameliorated by the administration of C1 and C1a. The level of inflammatory mediators such as MPO (**f**) and CRP (**g**) and immunoglobulins IgA and IgG2a (**h**) in the serum were measured by ELISA. Data are means ± SEM of three independent experiments. Note: ^#^ *p* < 0.05 is a significant difference compared with control group; * *p* < 0.05 is a significant difference compared with DSS-induced IBD group.

The typical symptoms of IBD include losses of body weight and stool consistency and rectal bleeding [19]. We used a DSS-induced IBD model, a widely recognized experimental animal model of IBD, to investigate the beneficial effects of C1 and C1a in IBD [33]. During the 2 weeks of DSS administration, the mice exhibited a significant loss of body weight and an increase in DAI score. However, these symptoms were ameliorated by the administration of C1 and C1a (Figure 2a,b).

Shortness of the intestine, including the colon and cecum, and splenomegaly are symptoms of intestinal inflammatory responses caused by immune cell activation [3]. The DSS-treated mice showed shortness of the colon and cecum (Figure 2c,d) and enlargement of the spleen (Figure 2e). However, the administration of C1a resulted in a recovery trend (Figure 2c–e). In particular, the C1a 5 mg/kg group showed a similar inhibitory effect at one-tenth of the dose of SS, the positive control drug.

The DSS-administered mice showed elevated serum MPO and CRP levels but showed an inhibitory trend following the administration of C1a (Figure 2f,g). The DSS-treated mice showed elevated serum IgA and IgG2a levels, which were alleviated by the administration of C1 and C1a (Figure 2h). These results indicated that C1 and C1a moderate IBD by suppressing inflammatory responses. In particular, it can be observed that the inhibitory effect of C1a is more effective than C1.

### 3.3. C1 and C1a Maintained the TJ by Suppressing Inflammatory Responses in a DSS-Induced IBD Mice Model

**Figure 3:**
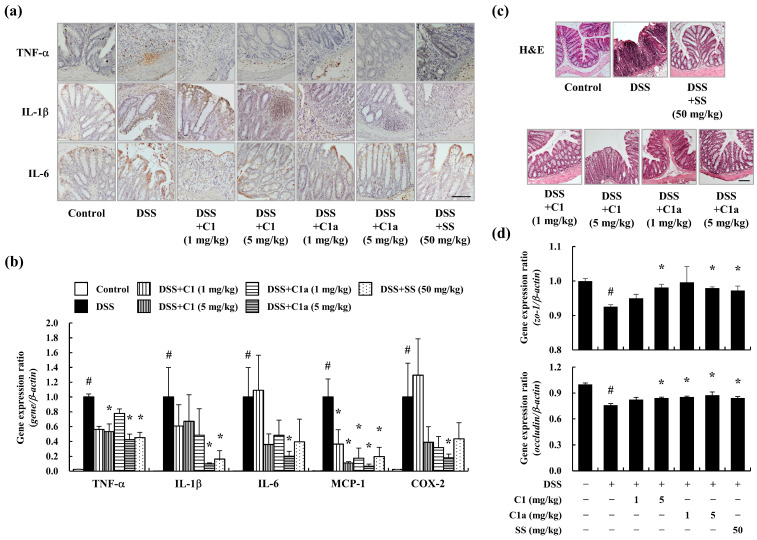
Effects of the FA derivatives C1 and C1a on anti-inflammation and barrier protection in a DSS-induced IBD mice model. A DSS-induced increase in pro-inflammatory cytokine expression in descending intestine tissues was assessed by IHC (**a**) and qPCR (**b**). Destruction of the epithelium barrier and intestinal structure was observed by H&E staining (**c**). A DSS-induced decrease in TJ protein levels was recovered after the administration of C1 and C1a (**d**). The magnification levels of representative histological images are 200× (**a**) and 100× (**c**). Scale bar: 0.1 mm. Data are means ± SEM of three independent experiments. Note: ^#^ *p* < 0.05 is a significant difference compared with control group; * *p* < 0.05 is a significant difference compared with DSS-induced IBD group.

DSS-induced inflammation leads to abnormal structural changes and dysfunction in the intestine [20,33]. To verify the intestinal inflammation in DSS-induced IBD, IHC staining was performed on the descending colon tissue samples. The DSS administration increased the levels of the pro-inflammatory cytokines TNF-α, IL-1β, and IL-6 in the epithelium layer and lamina propria (Figure 3a). In addition, the gene expression of pro-inflammatory mediators such as TNF-α, IL-1β, IL-6, MCP-1, and COX-2 is also elevated by DSS administration (Figure 3b). However, the administration of C1 and C1a suppressed these effects (Figure 3a,b).

In healthy people, the mucosal layer of the intestine is composed of a surface epithelium, lamina propria, and crypt, which are linked by tight junction proteins, including ZO-1 and occludin. In contrast, patients with IBD show a strong inflammatory response, abnormal structure in the mucosal layer, and dysfunction of their absorption efficacy [15]. Therefore, the TJ protein levels can serve as indicators of IBD severity. The DSS-treated mice showed mucosal structural damage, immune cell infiltration into the lamina propria, crypt abscesses, and cryptitis (Figure 3c). Moreover, the expression of TJ genes decreased after DSS administration (Figure 3d). However, the administration of C1 and C1a maintained the structure of the epithelium, crypt, and lamina propria, as well as the TJ gene expression (Figure 3c,d).

### 3.4. C1 and C1a Maintained the TJ by Suppressing Inflammatory Responses In Vitro

**Figure 4:**
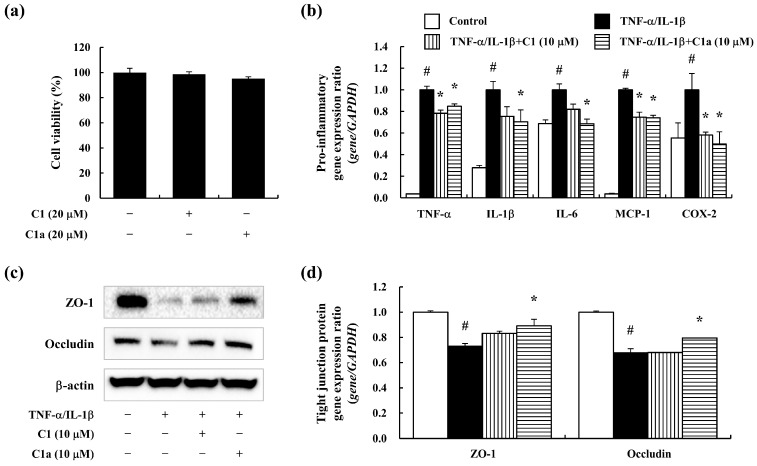
Effects of the FA derivatives C1 and C1a on anti-inflammation and barrier protection in TNF-α/IL-1β-stimulated Caco-2 cells: (**a**) the cytotoxicity of C1 and C1a was determined on Caco-2 cells; (**b**) the TNF-α/IL-1β-stimulated expression of pro-inflammatory cytokines such as TNF-α, IL-1β, IL-6, MCP-1, and COX-2 was analyzed by qPCR; the TNF-α/IL-1β-stimulated decrease in TJ protein levels such as ZO-1 and occludin was determined by Western blot (**c**) and qPCR (**d**). Data are means ± SEM of three independent experiments. Note: ^#^ *p* < 0.05 is a significant difference compared with control group; * *p* < 0.05 is a significant difference compared with TNF-α/IL-1β-treated group.

To evaluate the anti-inflammatory and barrier maintenance effects of C1 and C1a in vitro, a pro-inflammatory cytokine (TNF-α/IL-1β)-stimulated intestine epithelial cell line of Caco-2 cells was used. The C1 and C1a did not affect the survival rate of the Caco-2 cells up to 20 μM (Figure 4a). The TNF-α/IL-1β-stimulated Caco-2 cells expressed pro-inflammatory mediators including TNF-α, IL-1β, IL-6, MCP-1, and COX-2 but were decreased by the pre-treatment of C1 and C1a (Figure 4b). Additionally, the TNF-α/IL-1β-stimulated Caco-2 cells showed downregulated TJ protein (Figure 4c) and expression (Figure 4d) levels, which were recovered by C1 and C1a. In particular, C1a exhibited greater efficacy than C1 at the same concentration. These results suggest that C1 and C1a maintain the intestinal barrier’s integrity by alleviating inflammation.

## 4. Discussion

The main treatments for IBD include pharmacotherapy with 5-aminosalicylate (5-ASA), glucocorticoid, sulfasalazine, and immunomodulators. However, these drugs have adverse effects including headache, nausea, vomiting, gastric discomfort, diarrhea, immunodeficiency, pulmonary symptoms, and skin rashes [36,37]. Thus, there is growing interest in utilizing plant extracts that offer both safety and effectiveness in the treatment of IBD. Previous studies have demonstrated the protective anti-inflammatory effects of *P. oleracea* ethanol extracts on DSS-induced IBD [24,25]. In particular, the major compound of *P. oleracea*, ferulic acid, has been demonstrated to have various activities, such as anti-oxidant, anti-inflammatory, anti-Alzheimer’s, anti-cancer, cardioprotective, and also anti-IBD activities [12,26,27,28,30,34,38]. *N*-(*E*)-feruloyloctopamine (C1) is another compound isolated from *P. oleracea* that has anti-apoptotic [39], anti-oxidant [40], and anti-cancer [41] effects; however, its impact on IBD remains unexplored. Moreover, we modified and synthesized an FA derivative, C1a, to enhance its effectiveness and safety. In the present study, we investigated the therapeutic efficacy of C1 and C1a in IBD, while focusing on inflammation and intestinal barrier tight junctions.

Macrophages are the main effector cells involved in the development of IBD [42]. Although the cause of IBD is unclear, uncontrolled immune responses and hypersensitivity reactions have been reported [3]. When external factors such as pathogens penetrate the weakened intestinal barrier, macrophages are stimulated to produce inflammatory mediators, leading to damage and inflammation in the intestine [10]. Previous studies have demonstrated that pro-inflammatory mediators expressed by macrophages, such as NO, MCP-1, iNOS, and COX-2, contribute to the exacerbation of IBD, resulting in mucosal inflammation [43,44,45,46]. The major sources of MCP-1 are epithelial and immune cells, although MCP-1 is also produced by activated macrophages [47], which aggravate immune responses [48]. Our in vitro results showed that the pre-treatment with C1 and C1a inhibited NO release and pro-inflammatory mediators by suppressing MCP-1, iNOS, COX-2, and NF-κB activation in mouse macrophages. These results indicated that C1 and C1a have anti-inflammatory effects. Moreover, the activity level of C1a is higher than that of C1, thereby indicating its potential therapeutic efficacy in other inflammatory diseases. 

In this study, an animal model was established to evaluate the therapeutic effects of C1 and C1a in IBD. Many studies have shown that the severity of IBD was worsened by high levels of inflammatory cytokines such as IL-1β, IL-6, and TNF-α in patients [49]. Therefore, the identification of agents that modulate inflammatory cytokine production is crucial for the treatment of IBD. The DSS-induced mouse model exhibited various symptoms of IBD, such as weight loss, an increased DAI score, and shortening of the intestines, which were significantly ameliorated by the administration of C1 and C1a. In particular, the recovery of the colonic length was most prominent in the group treated with 5 mg/kg of C1a. In addition, inflammatory indicators such as increases in spleen weight and serum MPO, CRP, IgA, and IgG2a levels were ameliorated in a dose-dependent manner, especially at 5 mg/kg of C1a. This can be explained by the high anti-inflammatory efficacy of C1a, as confirmed by the in vitro analysis. Moreover, following this effect, the administration of C1 and C1a downregulated the levels of pro-inflammatory cytokines (TNF-α, IL-1β, and IL-6) and mediators (MCP-1 and COX-2) in the intestine. In particular, the inhibitory effects of C1a on pro-inflammatory cytokines and mediators were superior to those of sulfasalazine (at 10 times the dose). Our results suggested that C1a ameliorated intestinal inflammation.

To develop drug candidates with both anti-inflammatory and barrier-protective effects for the treatment of IBD, we conducted TJ-associated protein experiments. In our study, C1 and C1a allowed the recovery of the epithelial layer, crypt structure, and lamina propria. Furthermore, C1 and C1a maintained the levels of TJ-associated proteins, such as ZO-1 and occludin. The intestinal barrier plays a defensive role against inflammation [11]. In recent studies, patients with IBD have shown decreased TJ-associated protein expression and increased intestinal permeability due to reduced epithelial barrier function [14]. Strengthening the intestinal TJ provides an effective barrier to the external environment and is crucial for recovery from inflammatory bowel disease [11,12,50]. Our results indicated that the administration of C1 and C1a restored TJ-associated gene expression and protein levels caused by DSS administration. In addition, the barrier-protective effects of the C1 and C1a pre-treatment were confirmed through an in vitro analysis using Caco-2 cells. In particular, C1a exhibited greater efficacy than C1 at the same concentration. Our results suggest that C1 and C1a protect the barrier between the epithelial layer and the structure of the intestine by maintaining TJ protein expression.

## 5. Conclusions

In conclusion, this study demonstrated that compounds obtained from ferulic acid derivatives could prevent and exert therapeutic effects on IBD. C1 and C1a effectively inhibit inflammatory mediators, including through the production of pro-inflammatory cytokines and chemokines, which are responsible for inflammatory reactions. Furthermore, C1 and C1a prevented the destruction of the intestinal structure by maintaining the expression. These results demonstrated that C1 and C1a could be therapeutic candidates for the treatment of IBD. Moreover, because the beneficial effect of C1a was greater than for C1 and SS on inflammatory responses in vitro and in vivo, the effects of C1a should be evaluated in inflammatory diseases.

## Data Availability

The data presented in this study are available upon request from the corresponding author.

## Supplementary Materials

The following information can be downloaded from: https://www.mdpi.com/article/10.3390/toxics12040268/s1. Table S1: Primer sequences for qPCR. Table S2: Disease activity index. Figure S1: Synthesis and structures of ferulic acid (FA) derivatives (C1 and C1a). Figure S2: ESI-MS spectrum of C1. Figure S3: ^1^H NMR (600 MHz, methanol-*d*_4_) spectrum of C1. Figure S4: ^13^C NMR (150 MHz, methanol-*d*_4_) spectrum of C1. Figure S5: HRESIMS spectrum of C1a. Figure S6: ^1^H NMR (600 MHz, DMSO-*d*_6_) spectrum of C1a. Figure S7: ^13^C NMR (150 MHz, DMSO-*d*_6_) spectrum of C1a. Figure S8: DEPT-90 (150 MHz, DMSO-*d*_6_) spectrum of C1a. Figure S9: DEPT-135 (150 MHz, DMSO-*d*_6_) spectrum of C1a. Figure S10: COSY (600 MHz, DMSO-*d*_6_) spectrum of C1a. Figure S11: HMQC (600 MHz, DMSO-*d*_6_) spectrum of C1a. Figure S12: HMBC (600 MHz, DMSO-*d*_6_) spectrum of C1a. Figure S13: NOESY (600 MHz, DMSO-*d*_6_) spectrum of C1a. Figure S14. Key COSY, HMBC, and NOESY correlation of C1a.
