# Supplementary material for: Ferulic Acid Derivatives Ameliorate Intestine Barrier Destruction by Alleviating Inflammatory Responses in Dextran Sulfate Sodium-Induced Inflammatory Bowel Disease
Source: Toxics. 2024 Apr 3;12(4):268. doi: 10.3390/toxics12040268 (PMC11055104; doi:10.3390/toxics12040268)
Supplement: Supplementary file 1 [file toxics-12-00268-s001.zip › toxics-2937636-supplementary.pdf]

toxics

Supplementary Materials

Ferulic Acid Derivatives Ameliorate Intestine Barrier Destruction by Alleviating Inflammatory Responses in Dextran Sulfate Sodium-Induced Inflammatory Bowel Disease

Yeon-Yong Kim [1,†], Gayeong Hur [1,†], Hyun-Jae Jang [2,†], Seungwon Jeong [1], Seung Woong Lee [1], Seung-Jae Lee [1], Mun-Chual Rho [1], Sang-Hyun Kim [3,*] and Soyoung Lee [1,*]

Table S1. The primer sequence for qPCR.

Gene	Origin	Forward (5'- to -3')	Reverse (5'- to -3')
TNF-α	Mouse	AAGCCTGTAGCCCACGTCGTA	GGCACCACTAGTTGGTTGTCTTTG
IL-1β	Mouse	ATAACCTGCTGGTGTGTGAC	AGGTGCTGATGTACCAGTTG
IL-6	Mouse	CCACTTCACAAGTCGGAGGCTTA	GCAAGTGCATCATCGTTGTTCATAC
MCP-1	Mouse	CAGCAGGTGTCCCAAAGAA	CTTGAGGTGGTTGTGGAAAA
iNOS	Mouse	GTTCTCAGGCCAACAATACAAGA	GTGGACGGGTCGATGTCAC
COX-2	Mouse	GCCAGGCTGAACTTCGAAACA	GCTCACGAGGCCACTGATACCTA
ZO-1	Mouse	GGGGCCTACACTGATCAAGA	TGGAGATGAGGCTTCTGCTT
Occludin	Mouse	ACGGACCCTGACCACTATGA	TCAGCAGCAGCCATGTACTC
β-actin	Mouse	TAGACTTCGAGCAGGAGATG	TTGATCTTCATGGTGCTAGG
TNF-α	Human	GAGCTGAGAGATAACCAGCTGGTG	CAGATAGATGGGCTCATACCAGGG
IL-1β	Human	AAGTACCTGAGCTCGCCAGTG	GTGGTCGGAGATTCGTAGCTG
IL-6	Human	GGCACTGGCAGAAAACAACC	GCAAGTCTCCTCATTGAATCC
MCP-1	Human	GCAGAGGCTCGCGAGCT	ACAATGGTCTTGAAGATCACAGC
COX-2	Human	CCTGTGTTCCACCAGGAGAT	CCCTGGCTAGTGCTTCAGAC
ZO-1	Human	CAACATACAGTGACGCTTCACA	CACTATTGACGTTTCCCCACTC
Occludin	Human	ACAAGCGGTTTTATCCAGAGTC	GTCATCCACAGGCGAAGTTAAT
GAPDH	Human	GGAGCGAGATCCCTCCAAAAT	GGCTGTTGTCATACTTCTCATGG

Table S2. Disease activity index.

< Disease Activity Index >			
Score	Wight loss	Stool consistency	Bleeding
0	no loss	normal	no
1	1-5%		hemoccult positive
2	6-10%	loose stool	hemoccult positive and visual pellet bleeding
3	11-20%		
4	≥20%	diarrhea	gross bleeding

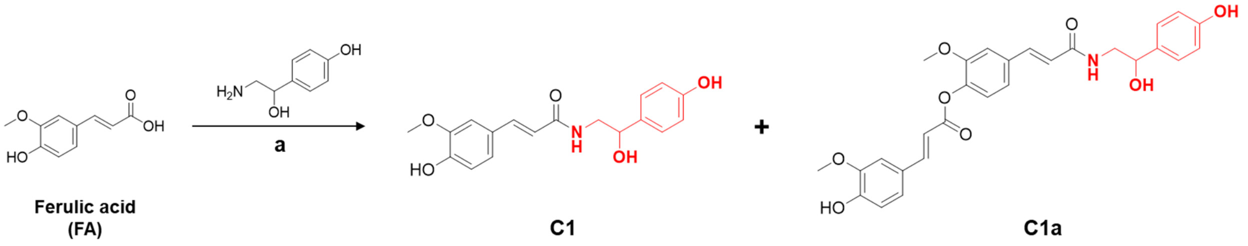

Figure S1. Synthesis and structures of ferulic acid (FA)-derivatives, (C1 and C1a). Reagents and conditions: (a) (±)-octopamine HCl, DMF soln. / stirring EDC for 10 min at -5°C, and then TEA stirring for overnight at room temperature.

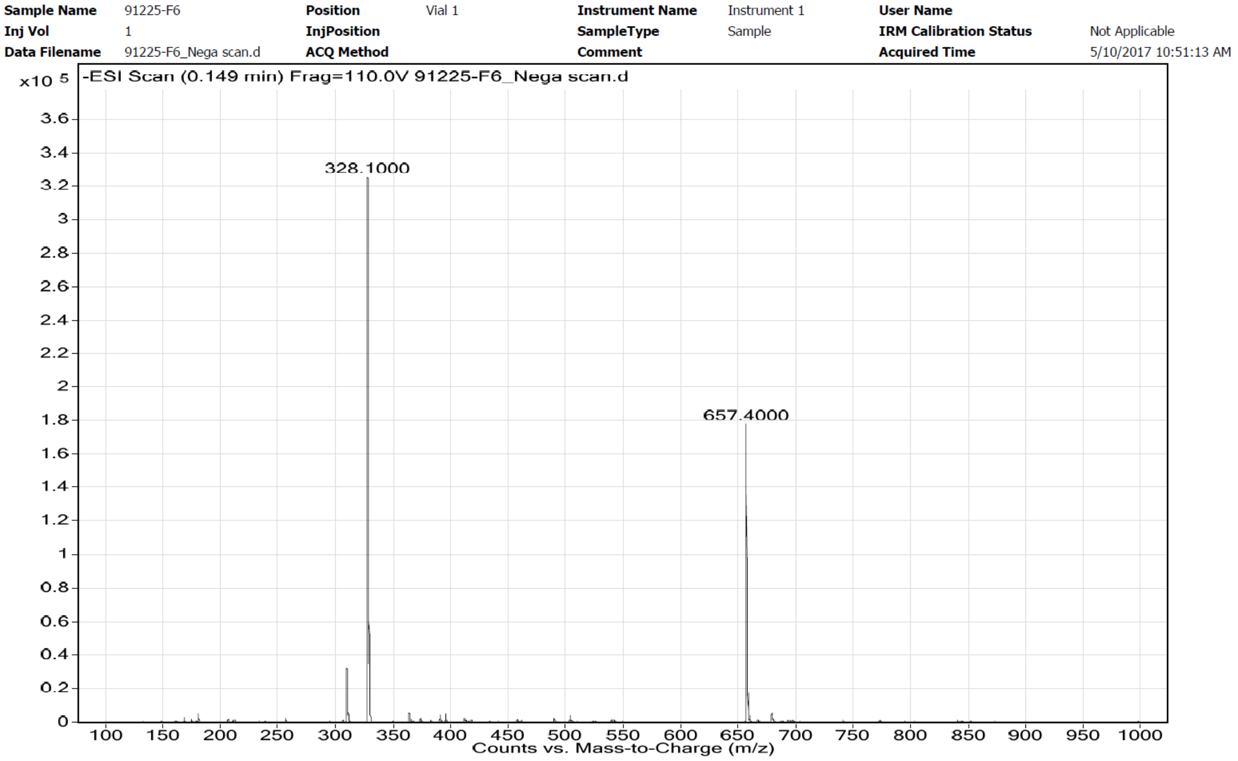

Figure S2. ESI-MS spectrum of C1.

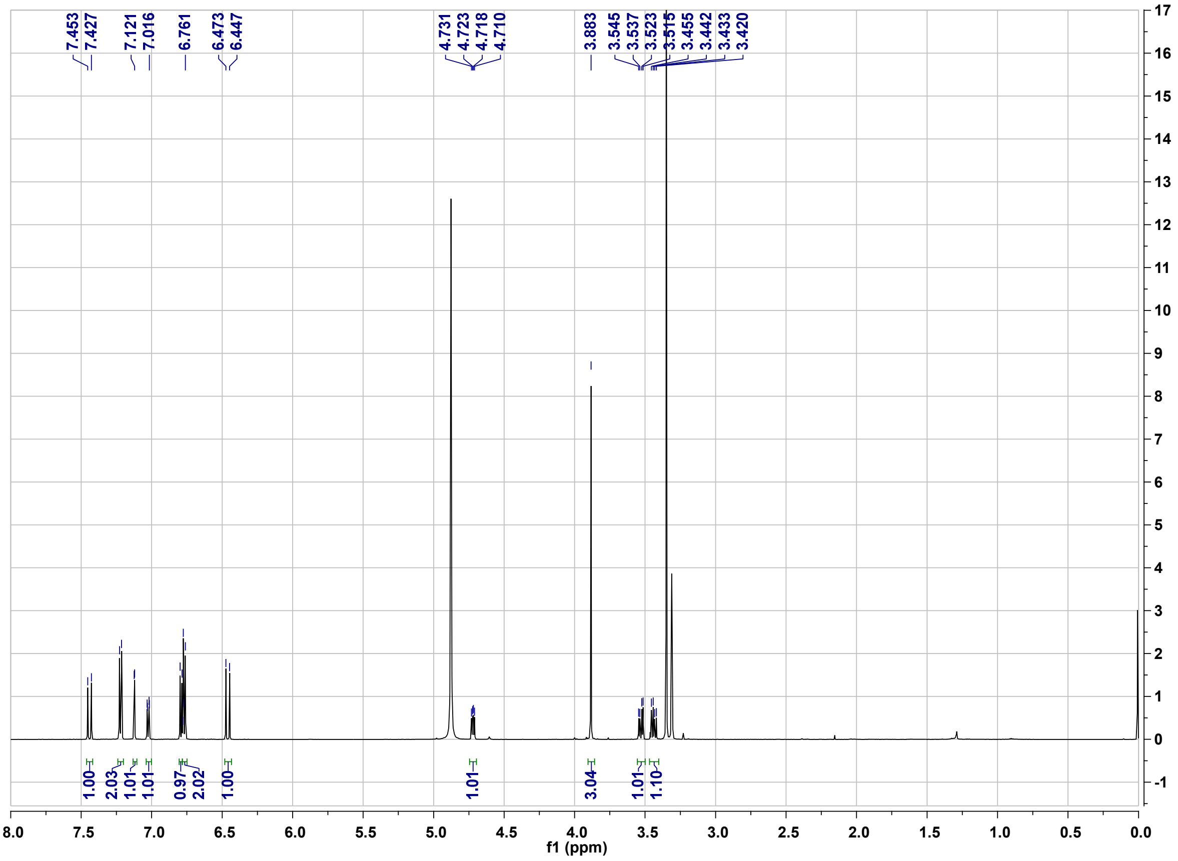

Figure S3. ^{1}H NMR (600 MHz, methanol-d_4) spectrum of C1.

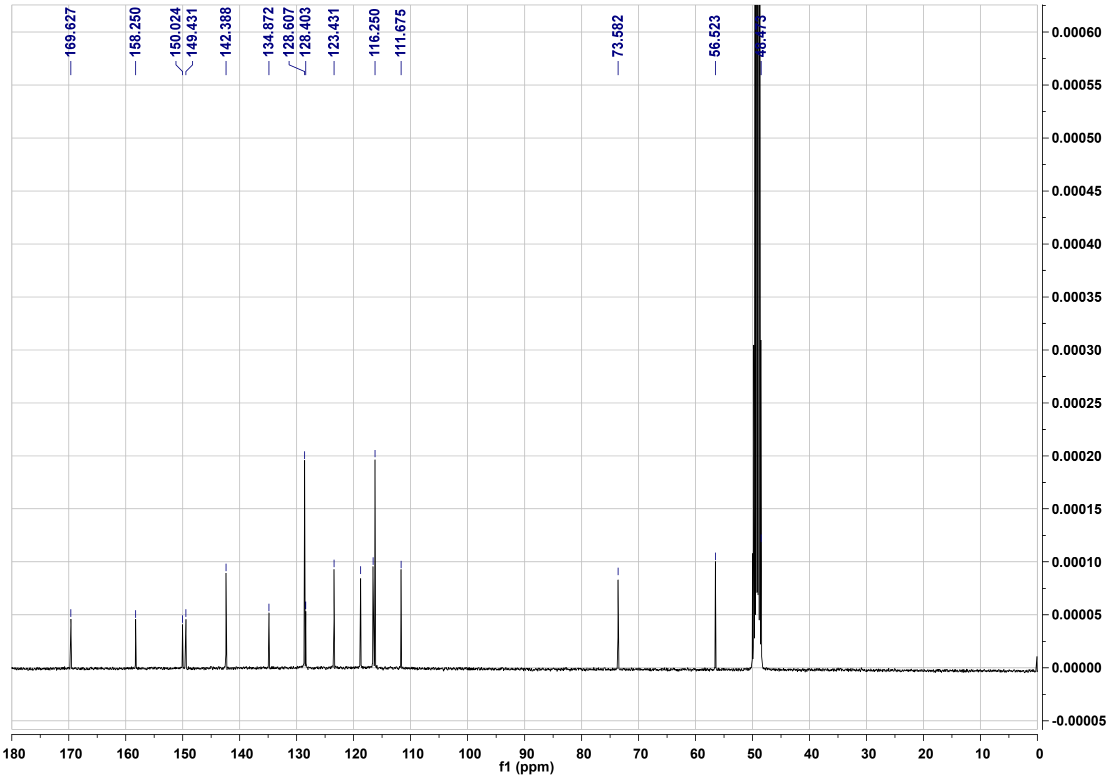

Figure S4. ^{13}C NMR (150 MHz, methanol-d_4) spectrum of C1.

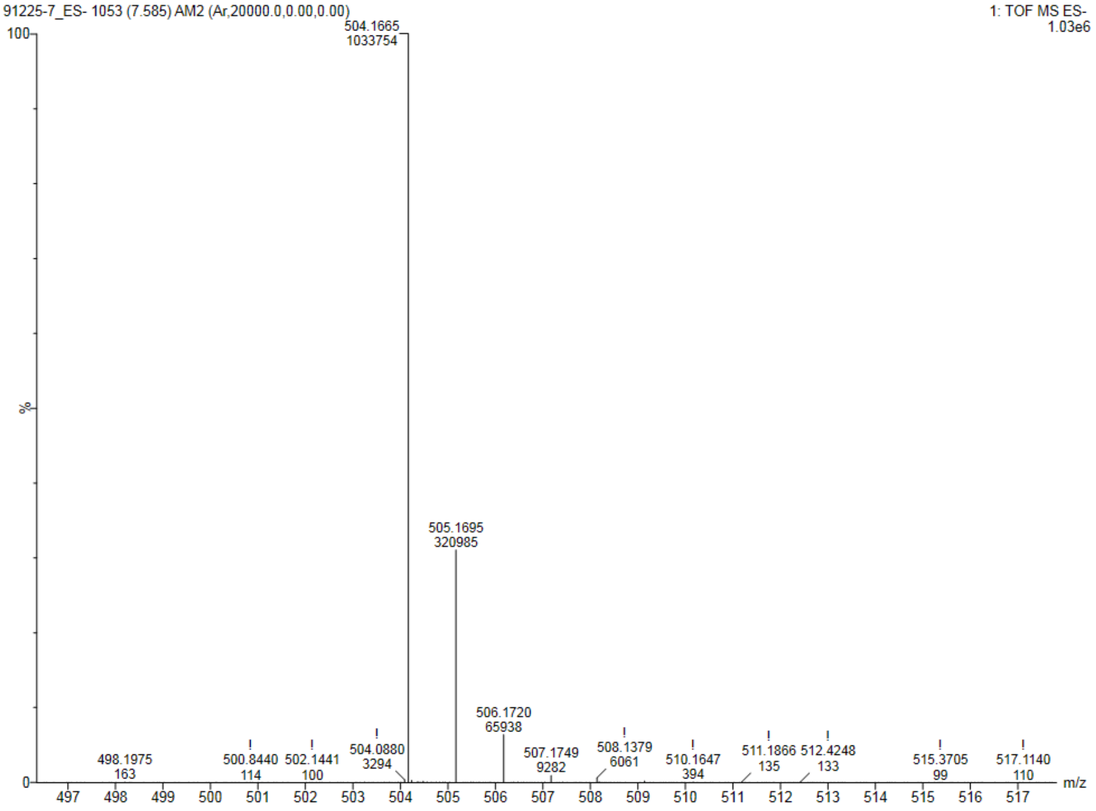

Mass	Calc. Mass	mDa	PPM	DBE	Formula	C	H	N	O
504.1665	504.1663	0.2	0.4	9.5	C13 H22 N13 O9	13	22	13	9
	504.1658	0.7	1.4	16.5	C28 H26 N O8	28	26	1	8

Figure S5. HRESIMS spectrum of C1a.

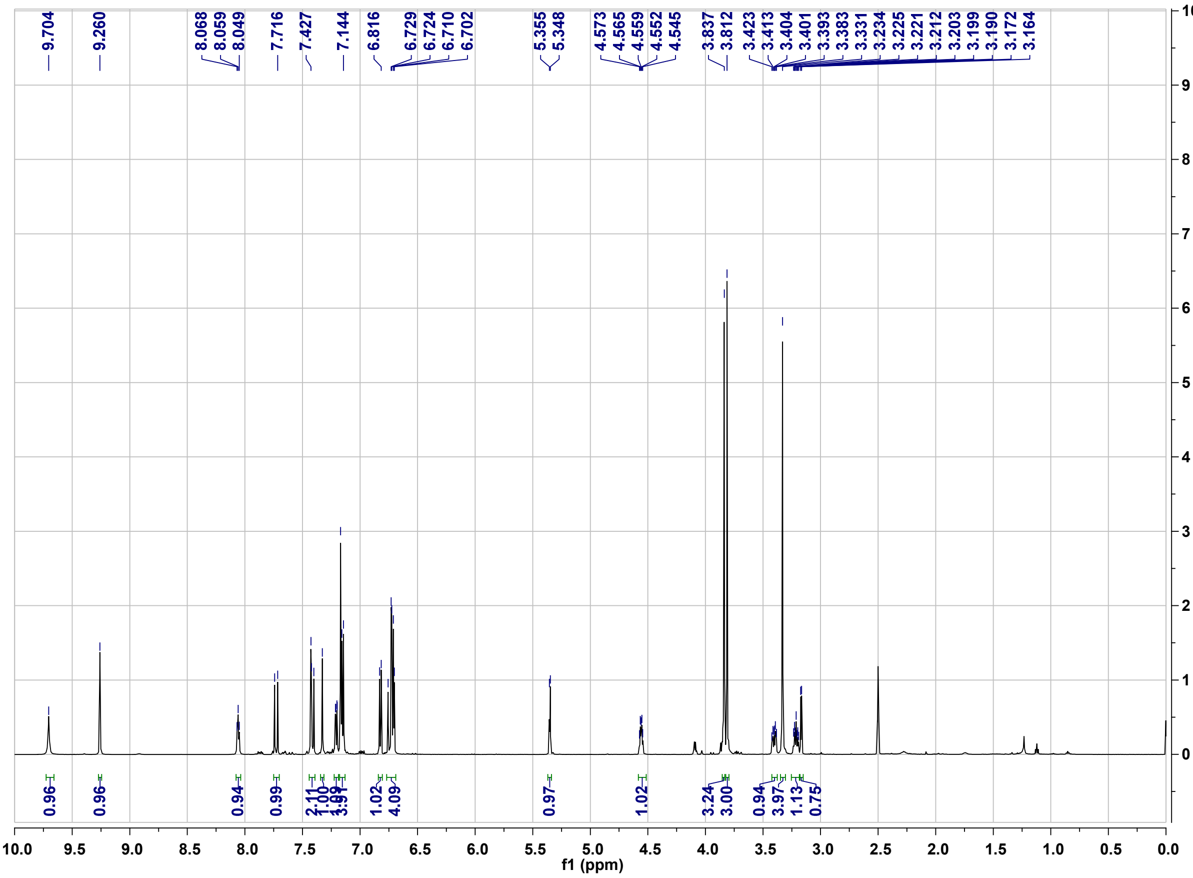

Figure S6. ^{1}H NMR (600 MHz, DMSO-d_6) spectrum of C1a.

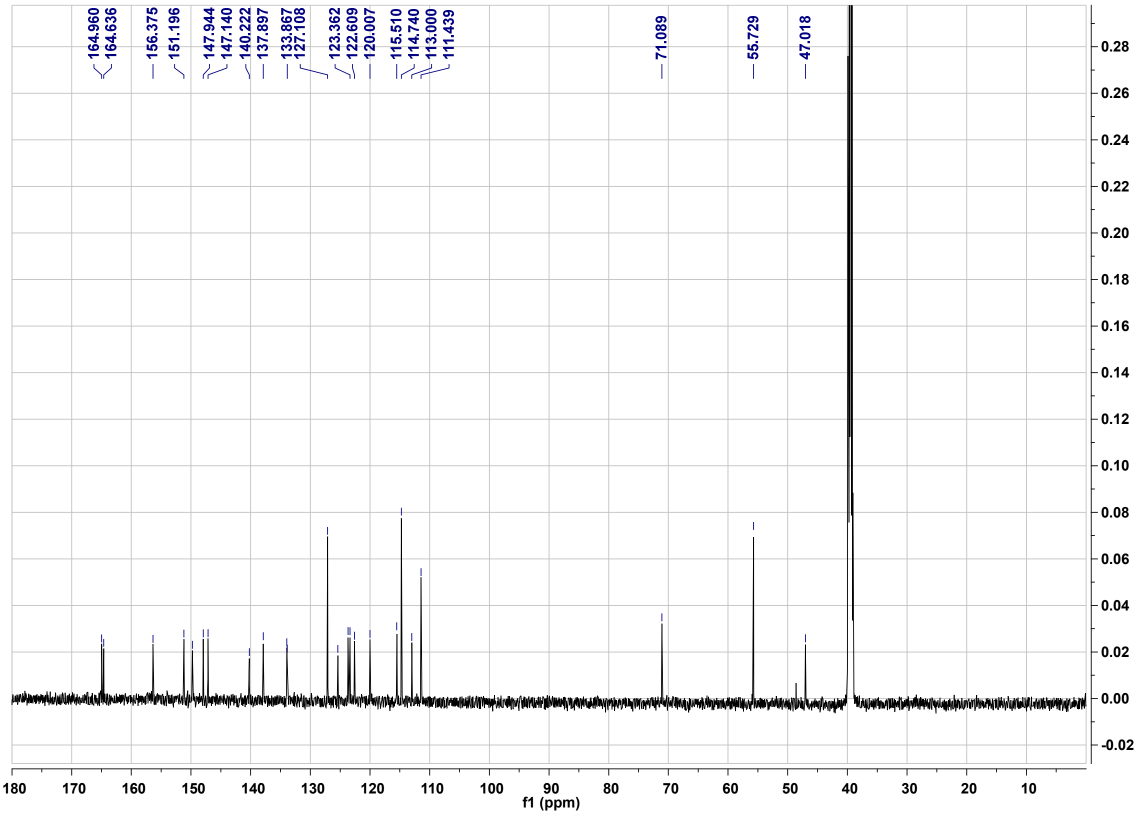

Figure S7. ^{13}C NMR (150 MHz, DMSO-d_6) spectrum of C1a.

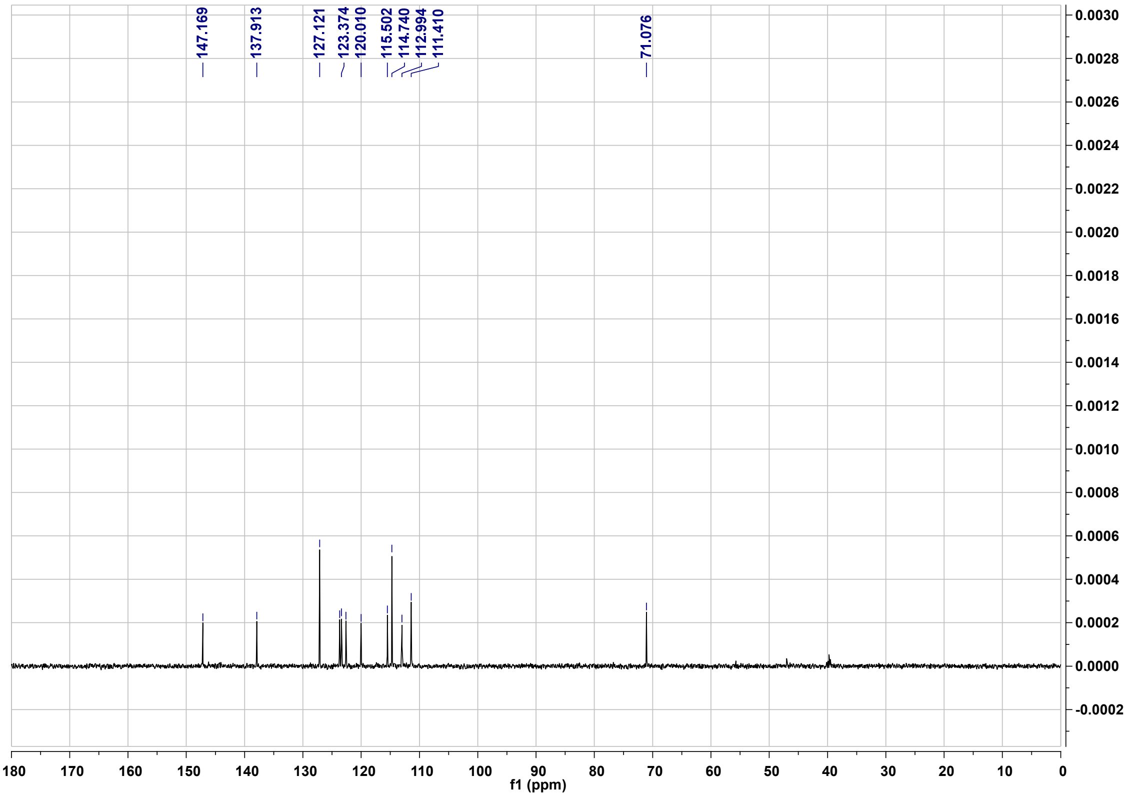

Figure S8. DEPT-90 (150 MHz, DMSO-d_6) spectrum of C1a.

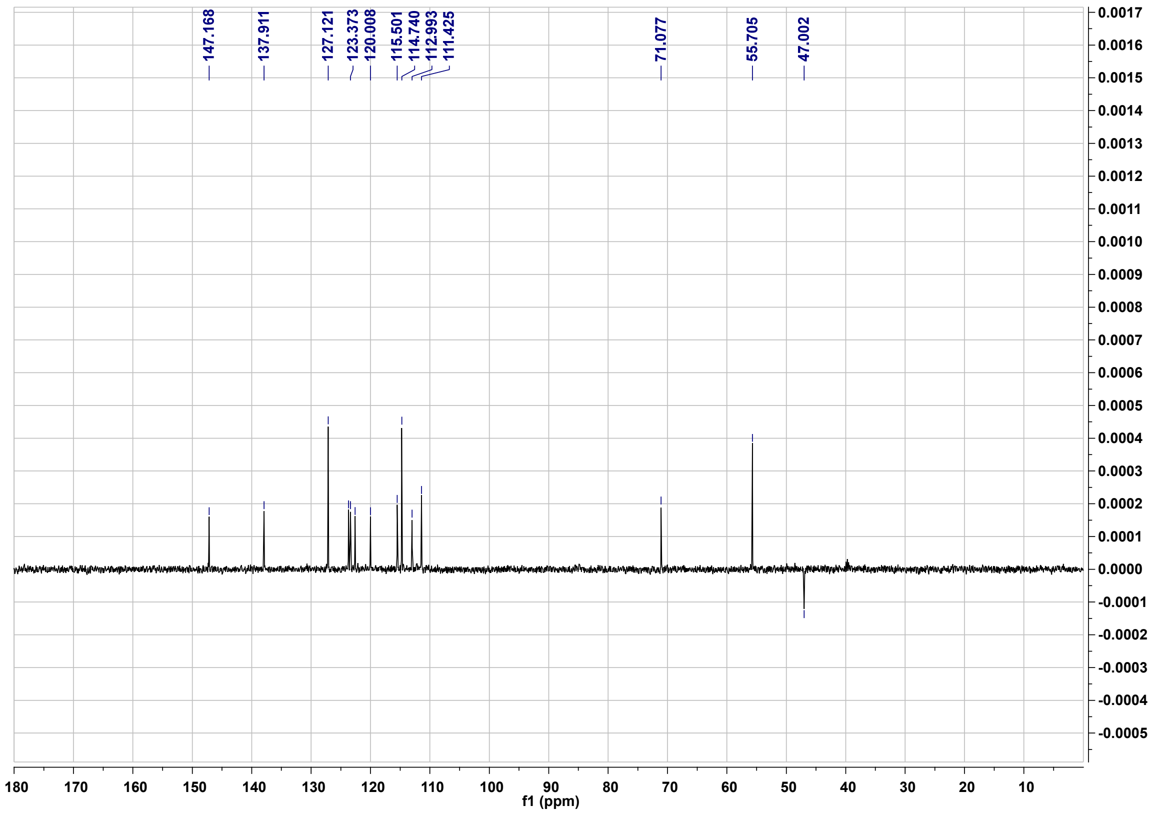

Figure S9. DEPT-135 (150 MHz, DMSO-d_6) spectrum of C1a.

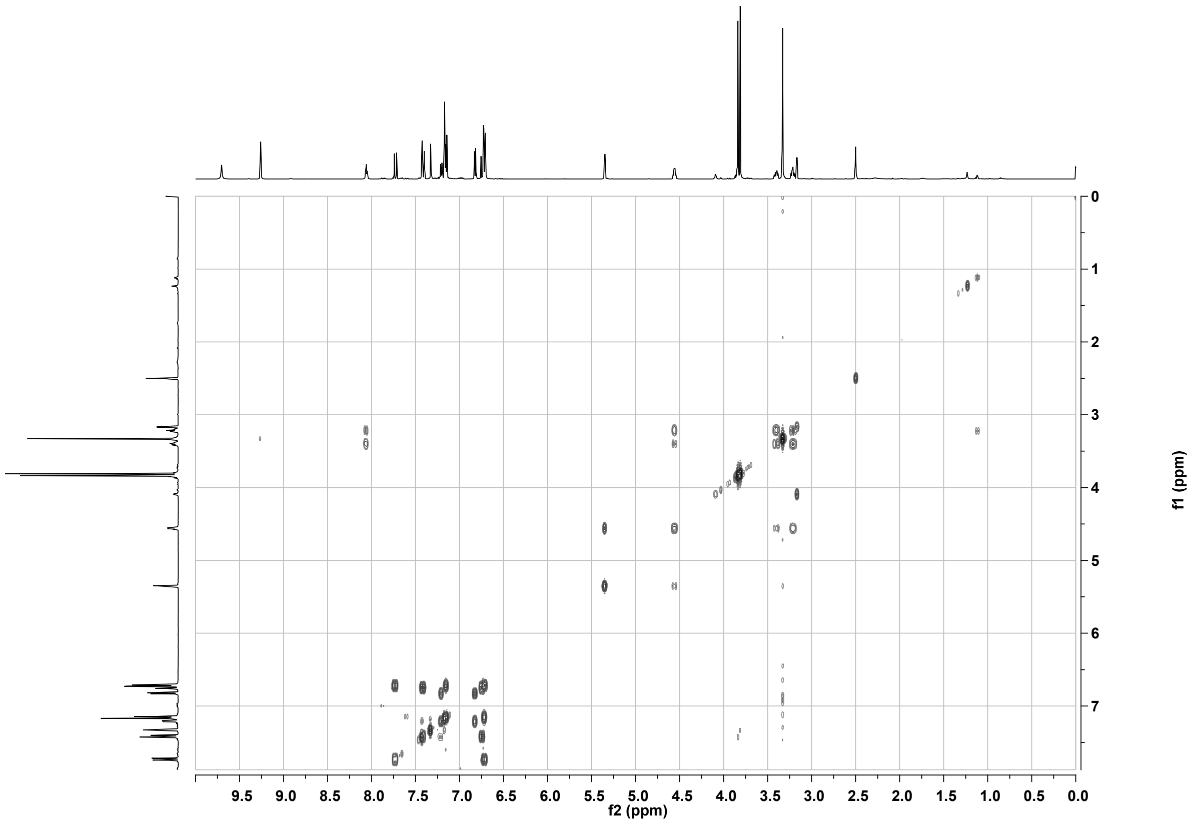

Figure S10. COSY (600 MHz, DMSO-d_6) spectrum of C1a.

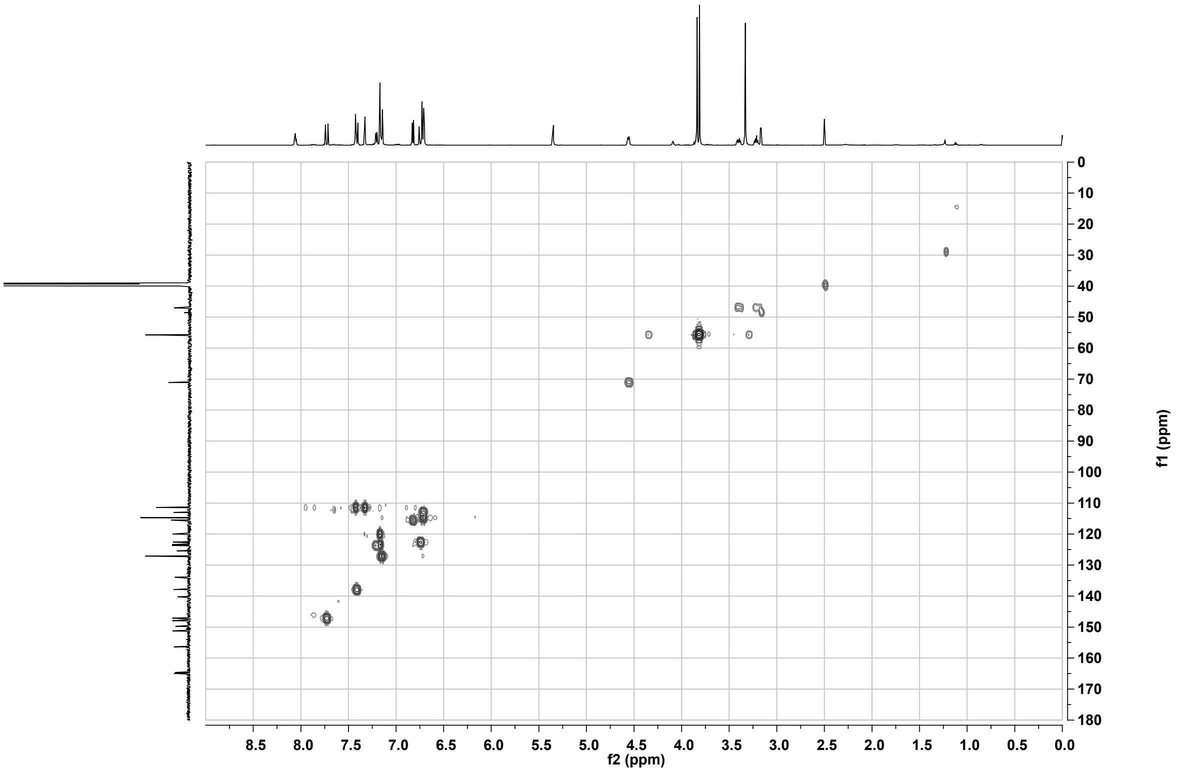

Figure S11. HMQC (600 MHz, DMSO-d_6) spectrum of C1a.

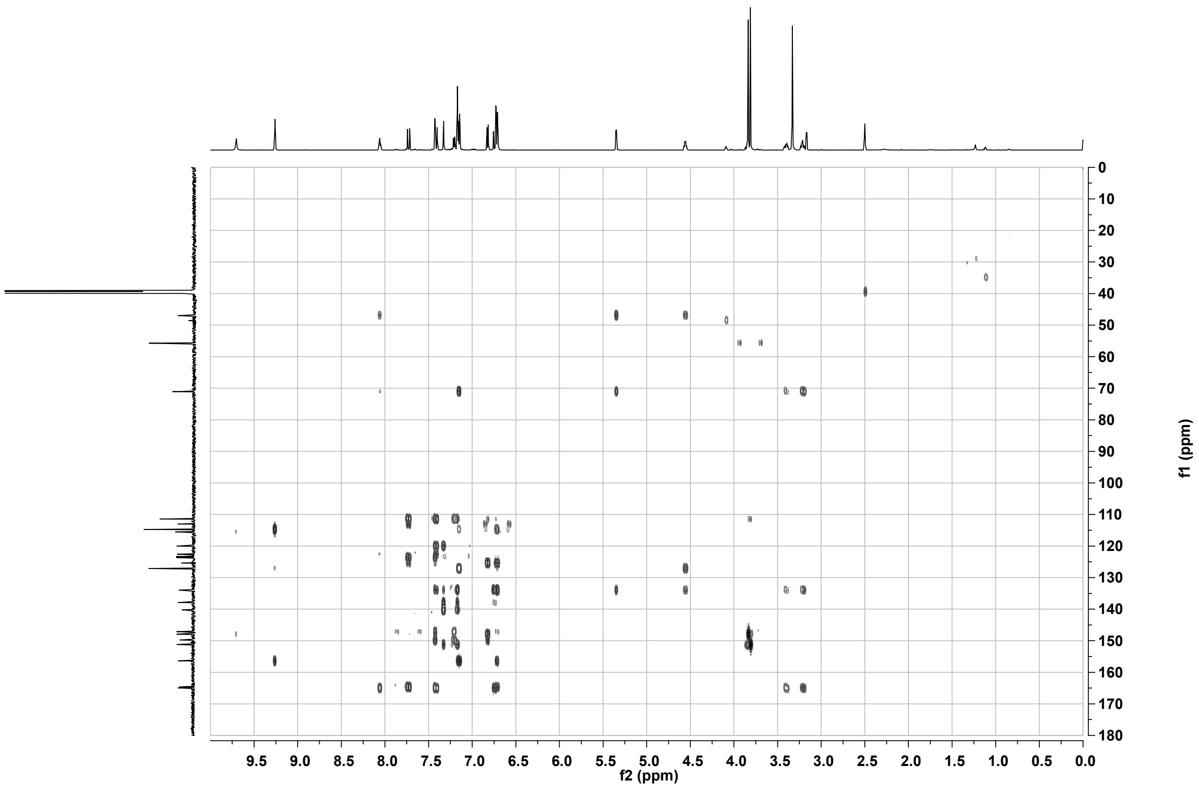

Figure S12. HMBC (600 MHz, DMSO-d_6) spectrum of C1a.

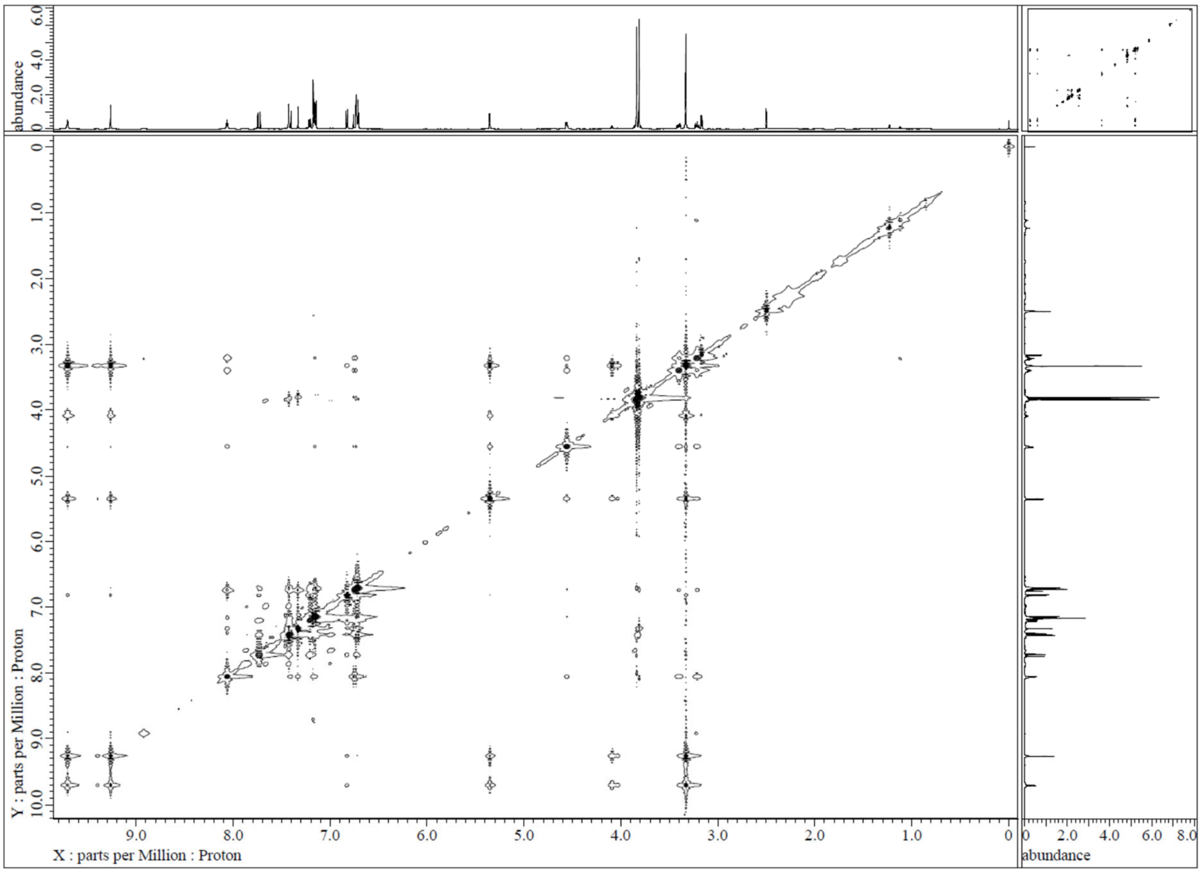

Figure S13. NOESY (600 MHz, DMSO-d_6) spectrum of C1a..

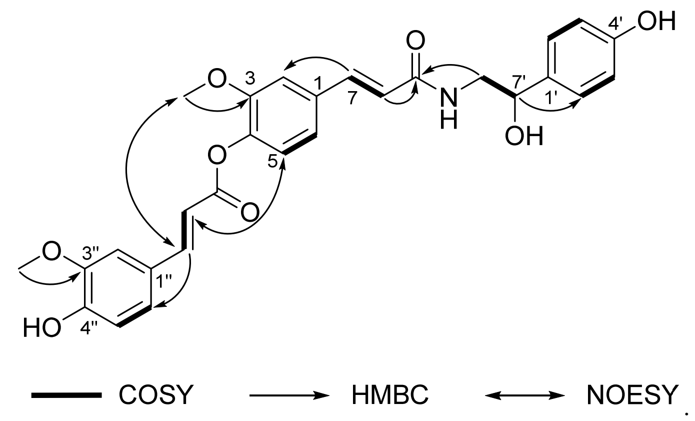

Figure S14. Key COSY, HMBC, and NOESY correlation of C1a.
